# Pulmonary microRNA profiling: implications in upper lobe predominant lung disease

**DOI:** 10.1186/s13148-017-0355-1

**Published:** 2017-05-30

**Authors:** David A. Armstrong, Amanda B. Nymon, Carol S. Ringelberg, Corina Lesseur, Haley F. Hazlett, Louisa Howard, Carmen J. Marsit, Alix Ashare

**Affiliations:** 1 Pulmonary and Critical Care Medicine, Dartmouth-Hitchcock Medical Center, Lebanon, NH USA; 2 Geisel School of Medicine at Dartmouth, Hanover, NH USA; 3 Department of Environmental Medicine and Public Health, Icahn School of Medicine at Mount Sinai, New York, NY USA; 4 Department of Environmental Health, Rollins School of Public Health, Emory University, Atlanta, GA USA

**Keywords:** MicroRNA, Epigenetics, Hypoxia, Alveolar macrophages, Exosomes, Microvesicles, Bronchoalveolar lavage

## Abstract

**Background:**

Numerous pulmonary diseases manifest with upper lobe predominance including cystic fibrosis, smoking-related chronic obstructive pulmonary disease, and tuberculosis. Zonal hypoxia, characteristic of these pulmonary maladies, and oxygen stress in general is known to exert profound effects on various important aspects of cell biology. Lung macrophages are major participants in the pulmonary innate immune response and regional differences in macrophage responsiveness to hypoxia may contribute in the development of lung disease. MicroRNAs are ubiquitous regulators of human biology and emerging evidence indicates altered microRNA expression modulates respiratory disease processes. The objective of this study is to gain insight into the epigenetic and cellular mechanisms influencing regional differences in lung disease by investigating effect of hypoxia on regional microRNA expression in the lung.

All studies were performed using primary alveolar macrophages (*n* = 10) or bronchoalveolar lavage fluid (*n* = 16) isolated from human subjects. MicroRNA was assayed via the NanoString nCounter microRNA assay.

**Results:**

Divergent molecular patterns of microRNA expression were observed in alternate lung lobes, specifically noted was disparate expression of miR-93 and miR-4454 in alveolar macrophages along with altered expression of miR-451a and miR-663a in bronchoalveolar lavage fluid. Gene ontology was used to identify potential downstream targets of divergent microRNAs. Targets include cytokines and matrix metalloproteinases, molecules that could have a significant impact on pulmonary inflammation and fibrosis.

**Conclusions:**

Our findings show variant regional microRNA expression associated with hypoxia in alveolar macrophages and BAL fluid in the lung—upper vs lower lobe. Future studies should address whether these specific microRNAs may act intracellularly, in a paracrine/endocrine manner to direct the innate immune response or may ultimately be involved in pulmonary host-to-pathogen trans-kingdom cross-talk.

**Electronic supplementary material:**

The online version of this article (doi:10.1186/s13148-017-0355-1) contains supplementary material, which is available to authorized users.

## Background

Numerous pulmonary diseases occur with upper lobe predominance including cystic fibrosis (CF), smoking-related chronic obstructive pulmonary disorder (COPD), tuberculosis, and sarcoidosis [1]; however, the initiating molecular events of the regional inflammatory response in these diseases have yet to be determined.

In the healthy lung, a physiological hypoxic oxygen gradient exists from the apex (21% O_2_) to the base (10–11% O_2_) of the lung in the upright position [2, 3]. Physiological factors such as lymphatic flow, as well as metabolic factors such as uptake of O_2_, elimination of CO_2_, and pH of airway surface liquid, differ regionally within the lung due to inequalities of the ventilation-perfusion ratio [1]. Hypoxia is known to exert profound effects on various important aspects of cell biology, for example, expression of cell surface markers, cell viability, phagocytosis, metabolic activity, and cytokine release [4, 5].

Alveolar macrophages (AM) are immune cells that play a pivotal role in the detection and elimination of pathogenic microorganisms [6]. AMs are one of multiple cell types, including type I and type II epithelial cells, endothelial cells and fibroblasts that reside within the lung alveoli [7]. Macrophages are phagocytic cells and crucial effectors of innate immunity in the primary response to pathogens besides their key role in acute and chronic inflammatory responses. Many pathological processes with macrophage involvement (e.g., inflammation, wound healing, atherosclerosis, and tumors) are characterized by hypoxia. As macrophages are of paramount importance in the first line defense against invasive microorganisms, it has long been hypothesized that these cells must be especially equipped to cope with and function in hypoxic areas [4].

Epigenetics is the study of heritable changes in gene function caused by mechanisms other than changes in the underlying DNA sequence [8]. One important mode of epigenetic regulation is expression of microRNAs (miRNA) [9]. MicroRNAs are ubiquitous regulators of human biology [10] and increasingly being recognized as important modulators in respiratory disease [11, 12]. MicroRNAs are a class of non-coding RNAs of 19 to 24 nucleotides in length that regulate gene expression through post-transcriptional, RNA interference, and gene silencing pathways. Initially thought to act primarily intracellularly, circulating miRNAs have gained attention as extracellular messengers and have been found associated with actively secreted exosomes/microvesicles and HDL particles [13, 14]. The number of annotated human microRNA loci currently numbers at more than 2500 in the latest version of miRBase (v.21) [15]. Functionally, in the context of cell-to-cell communication in disease, by working to fine-tune protein expression levels, miRNAs can contribute to regulatory circuits by providing quantitative control of gene output. In particular, miRNAs potentially exert their influence by regulating dosage-sensitive genes for which small fluctuations in protein expression may contribute to a substantial functional output [16]. In addition to their role in host cell-to-cell communication, microRNAs have been implemented in an emerging field of study in higher eukaryotes referred to as trans-kingdom cross-talk, whereby RNA-signal exchange have been described to occur between organisms of different kingdoms [17]. This topic is currently underexplored in pulmonary disease.

Regional differences in resident lung macrophage and other cells’ responsiveness to hypoxia may be an important factor in the initial development of pulmonary diseases with upper lobe predominance. Hypoxia has been implicated as an important proximal regulator of miRNA biogenesis and function [18]. Furthermore, as miRNAs have emerged as important epigenetic regulators of the innate and adaptive immune response [19], we assessed microRNA content of upper lobe and lower lobe lung macrophages based on conditional hypoxia as well as upper lobe and lower lobe bronchoalveolar lavage (BAL) fluid for altered microRNA expression.

The objective of this study is to gain insight into the cellular mechanisms influencing pulmonary diseases occurring with upper lobe predominance, specifically understanding how hypoxia may impact the regional innate immune response and if microRNAs may in part be responsible.

## Methods

This study was approved by the Committee for the Protection of Human Subjects at the Geisel School of Medicine at Dartmouth (#22781). Following written informed consent, subjects underwent flexible bronchoscopy to obtain BAL fluid and primary alveolar macrophages (AM). This procedure has been previously described in detail [20]. Primary AM were isolated from BAL fluid via gravity filtration through 2 layers of gauze, followed by three sequential washes in normal saline with subsequent centrifugation to pellet cells (400 × g/5 min/20 °C). AM were resuspended in RPMI 1640 media (50 μg/ml glutamax/gentamicin) and equilibrated overnight in vitro (normoxic) and then subject to continued normoxic (21% O_2_) or hypoxic conditions (5% O_2_). Total RNA was extracted from AM using Zymo Quick-RNA Mini-prep Kit (Irvine, CA). Cell-free microRNA from BAL fluid was obtained using Norgen Urine Exosome RNA Isolation Kit (Norgen Biotek Corp., Thorold, ON, Canada) from a 0.3 ml volume of cell-free BAL fluid. All protocols were performed according to manufacturer’s instructions. The digital multiplexed NanoString nCounter human v3 microRNA expression assay (NanoString Technologies, Seattle, WA) was performed according to manufacturer’s instructions with total RNA or miRNAs extracted as above. Briefly, 3–10 ng microRNA (BAL fluid) or 100 ng total RNA (AM) samples were prepared by ligating a specific miR-tag onto the 3’ end of each mature miRNA followed by an overnight hybridization (65 °C) to nCounter Reporter and Capture probes. Excess Reporter and Capture probes are washed away using the automated nCounter sample prep station and probe/target complexes are aligned and immobilized in the nCounter Cartridge. Cartridges are then placed in the nCounter digital analyzer for data collection. nSolver Analysis software (NanoString) (V3.0) was used for data analysis including background correction by subtracting the mean of the six negative controls included on the NanoString platform and normalization using the average geometric mean of the top 100 probes detected. Additional methods including: total RNA/microRNA extraction/purification/quantification, droplet digital PCR, ELISAs, and electron microscopy are detailed in the on-line data supplement—methods section (Additional file 1). Bio-analyzer electropherograms for microRNA quantitation are presented in on-line Additional file 2: Figure S1 and NanoString technical replicates are show in on-line Additional file 3: Figure S2. Gene ontology (GO) and pathway analysis were performed using MirTarBase [21]. All data were presented as means ± SD. Graph Pad Prism (v7.0), *t* tests, one-way ANOVA with post-hoc Tukey-Kramer HSD (cytokine analysis) and Spearman correlations were used along with Partek Genomics Suite 6.6 for paired sample *t* test to determine differential microRNA expression.

## Results

### Patient demographics

A total of 16 healthy adult subjects (non-smokers) were enrolled in this study. The median age of the cohort was 27.94 (±4.28) years. Gender distribution was comprised of seven males and nine females.

### Divergent inflammatory response in alveolar macrophages from alternate lobes of the lung with oxygen stress

**Fig. 1:**
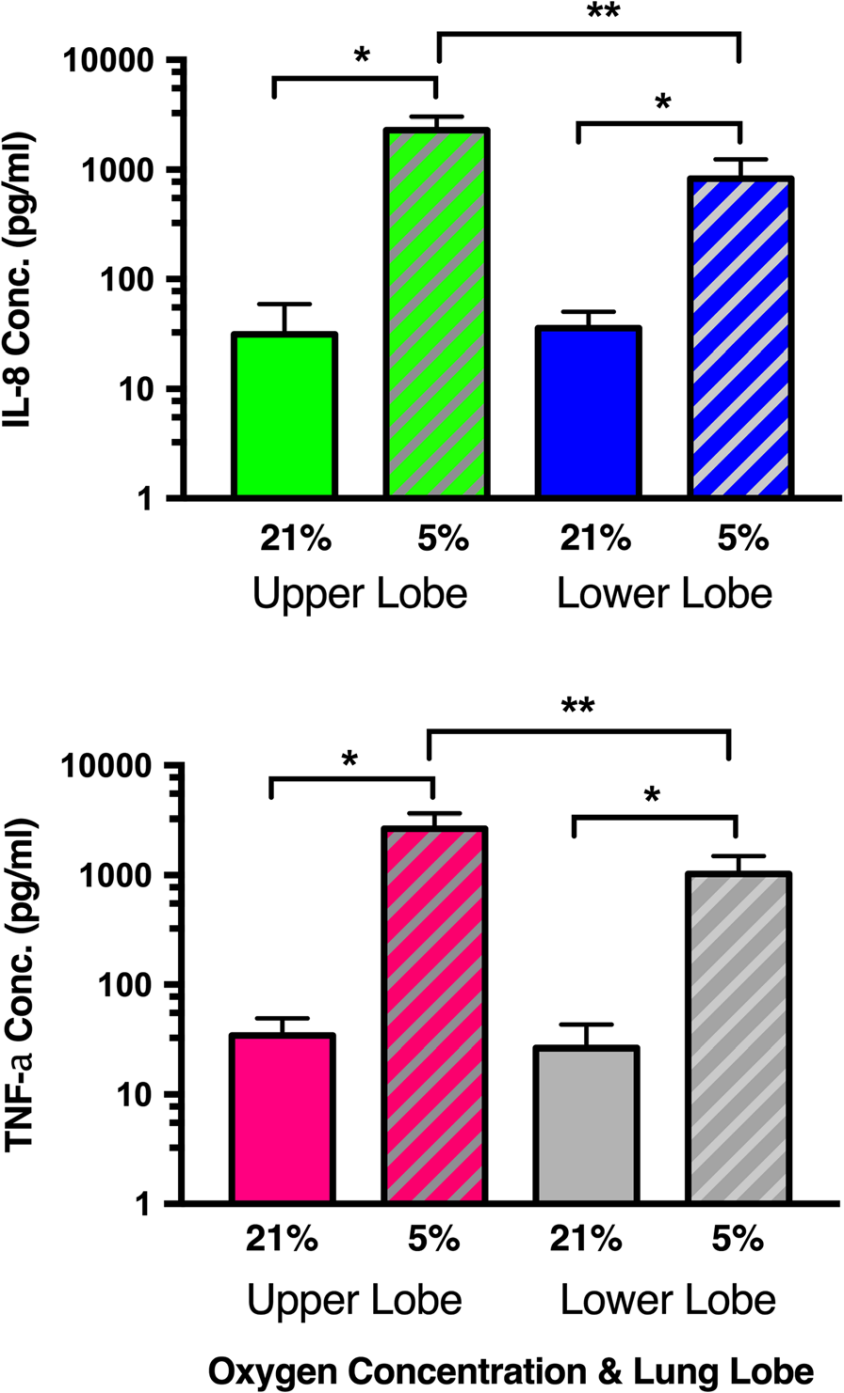
Secretion of inflammatory cytokines by regional alveolar macrophages in response to hypoxia. Alveolar macrophages obtained via bronchoalveolar lavage (*right upper and lower lobes*) were subject to in vitro hypoxic conditions (5% O_2_ for 60 min) in an in vivo_2_ 300 hypoxic chamber (Baker Ruskinn). Cytokines IL-8 and TNF-α (pg/ml) were measured by ELISA from conditioned media. Basal levels of cytokines secreted from AM are <35 pg/ml in 21% O_2_, however a significant increase in cytokine secretion occurs when macrophages are oxygen stressed at 5% O_2_ (ANOVA Tukey-Kramer HSD *P* < 0.0001). *Hypoxia induced increase in cytokine levels increase in both lobes (*P* < 0.0001). **Cytokine secretion was quantitatively higher from upper lobe AM (IL-8: upper 2294.6 (+731.8) vs. lower 830.4 (+415.8) pg/ml) (*P* < 0.0001). (TNF-α: upper 2656.9 (+986.8) vs. lower 1031.1 (+464.1) pg/ml) (*P* < 0.0001). Results from three independent experiments are reported as the means ± SD

Primary alveolar macrophages from the right upper lobe (RUL) and the right lower lobe (RLL) of the lung were obtained from healthy volunteers via bronchoalveolar lavage, equilibrated overnight in vitro (normoxic) and then subject to continued normoxic (21% O_2_) or hypoxic conditions (5% O_2_) for 1 h. Early response cytokines IL-8 and TNF-α were measured by ELISA from conditioned media. A significant increase in IL-8 and TNF-α secretion was detected from both upper lobe and lower lobe AM under oxygen stress (Fig. 1). Fold changes in cytokine secretion (up to 20×) were observed after just 60 minutes of in vitro hypoxia (5% Oxygen exposure) from upper lobe AM (IL8: 31 pg/ml to 2295 pg/ml) (ANOVA *P* < 0.0001), (TNFα: 34–2657 pg/ml) (*P* < 0.0001) and lower lobe AM (IL8: 31 pg/ml to 830 pg/ml *P* = 0.004) (TNFα: 35–1031 pg/ml) (*P* = 0.0051). Of particular note, illustrating a differential zonal response, lower lobe alveolar macrophages did not respond as prominently to hypoxia as did their upper lobe counterpart, consistent for both cytokines (IL8: Upper 2295 pg/ml vs. Lower 830 pg/ml (*P* < 0.0001) and TNFα: Upper 2657 pg/ml vs Lower 1031 pg/ml) (*P* < 0.0001).

### Hypoxia influences microRNA expression in alveolar macrophages

Regional changes in microRNA profiles and content were assayed in AM under in vitro normoxic (21% O_2_) or hypoxic oxygen stress conditions (5% O_2_ - 24 h). Of the 800 different microRNAs measureable with the NanoString nCounter miR assay platform, 73 microRNAs were detectable in AM samples. The top 10 highest expressed microRNAs were nearly identical in alveolar macrophages under all experimental conditions examined (Table 1).

**Table 1:** Top 10 microRNAs in alveolar macrophages (AM)

Gene name	Mean miRNA read counts^a^	SD
hsa-let-7a-5p	5113	1983
hsa-miR-23a-3p	3162	1354
hsa-let-7b-5p	2964	1133
hsa-miR-223-3p	2802	1105
hsa-miR-191-5p	2785	1348
hsa-miR-342-3p	1929	733
hsa-miR-4454	1090	1073
hsa-miR-21	962	440
hsa-miR-155	624	250
hsa-let-7d-5p	583	328

^a^Input: 100 ngs total RNA

This group of most highly expressed microRNAs from healthy lung macrophages include: three let-family members (let-7a-5p, let-7b-5p, and let-7d-5p), miR-23a, miR-223, miR-4454, and miR-155. Next, comparative analysis was done amongst microRNA profiles from the varying in vitro experimental conditions. First, we compared miRNA profiles from upper vs. lower AM under normoxic conditions. Log2 fold changes along with *P* values are shown in Table 2.

**Table 2:** Differentially expressed MicroRNAs comparing upper lung and lower lung AM

microRNA ID	Expression change	logFC	*P* value
Normoxia			
Upper vs. lower^a^			
hsa-miR-30e-5p	Increase	0.313	0.018
hsa-miR-4454	Decrease	−0.986	0.032
hsa-miR-4443	Decrease	−0.757	0.003
Hypoxia			
Upper lobe MO^b^			
hsa-miR-664a-3p	Decrease	−0.498	0.005
Lower lobe MO^b^			
hsa-miR-30e-5p	Increase	0.412	0.015
hsa-miR-93-5p	Increase	0.306	0.036
hsa-miR-4454	Decrease	−1.057	0.019
hsa-miR-22-3p	Decrease	−0.540	0.007

microRNA ID = official microRNA name according to miRBASE v21; logFC = log2 fold change in macrophages from upper lobe vs. lower lobe

*P* value = the unadjusted *P* value from the statistical test (paired analysis)

^a^Upper lobe AM vs. lower lobe AM

^b^21%O_2_ vs. 5% O_2_

Three microRNAs were significantly different between regional macrophages miR-4443 (*P* = 0.003), miR-30e-5p (*P* = 0.018) and miR-4454 (*P* = 0.032). Next, miRNA profiles from AM within each lobe were analyzed comparing normoxia to hypoxia. In the upper lobe, miR-664a-3p (*P* = 0.005) was the lone microRNA identified as significantly changed upon 24 h exposure to 5% O_2_. However, in the lower lobe, four microRNAs were significantly altered including: miR-22-3p (*P* = 0.007), miR-30e-5p (*P* = 0.015), miR-4454 (*P* = 0.019), and miR-93-5p (*P* = 0.036).

### MicroRNA profiling of BAL Fluid

**Fig. 2:**
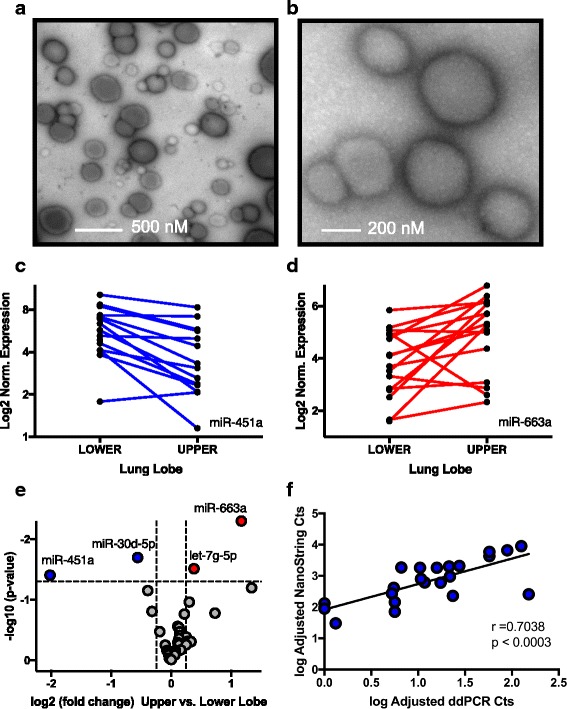
Divergent microRNA expression from isolated microvesicles/exosomes of BAL Fluid - Right Upper Lobe (RUL) vs. Right Lower Lobe (RLL). Microvesicles/exosomes in BAL fluid visualized via negative stain electron microscopy with a JEOL TEM1010 transmission electron microscope (**a** + **b**) (*scale bar* in nanometers). Individual microRNAs in BAL with highest log2 fold change RUL vs. RLL BAL fluid: miR-451a (**c**) (*P* = 0.039) & miR-663a (**d**) (*P* = 0.005). Each line represents directional change in individual samples (total *n* = 16). Volcano plot of microRNA expression differences (log2 fold change) from BAL samples (**e**). Scatterplot of ddPCR with Taqman assays and NanoString counts ofmicroRNA-451a, coefficient reported from Spearman correlation (**f**) (*rho* = 0.7038) (*P* < 0.003)

Lung fluid from both the right upper lobe and the right lower lobe was obtained by performing bronchoalveolar lavage on 16 healthy subjects. MicroRNA was isolated, purified and quantitated. Prior to NanoString miRNA assay, BAL fluid was evaluated for presence of exosomes /microvesicles by negative stain electron microscopy (Fig. 2a, b). Exosomes /microvesicle size range was 50–600 nm. Of the 800 different microRNAs measureable with the NanoString nCounter platform, 35 microRNAs were detectable in BAL samples. Table 3 lists the four microRNAs with significant differences in expression levels comparing upper lobe and lower lobe BAL, plus log2 fold change and *P* < 0.05, respectively.

**Table 3:** Differentially expressed MicroRNAs in upper lung vs. lower lung BAL fluid

Expression change			
microRNA ID	UL vs. LL	logFC	*P* value
hsa-miR-663a	Increase	1.17	0.005
hsa-let-7 g-5p	Increase	0.38	0.031
hsa-miR-451a	Decrease	−2.02	0.039
hsa-miR-30d-5p	Decrease	−0.56	0.020

microRNA ID = official microRNA name according to miRBASE v21; logFC = log2 fold change in BAL from upper lobe vs. lower lobe; *P* value = the unadjusted

*P* value from the statistical test (paired analysis)

Comparison of the changes from each BAL sample (*n* = 16) lower lobe vs. upper lobe log2 normalized expression of the two miRNAs with largest fold change are shown in Fig. 2 c, d. MiR-451a demonstrates reduced expression in upper compared to lower lobe BAL, while miR-663a shows higher expression. Additionally, the overall distribution of microRNA expression differences (log2-fold change) comparing upper to lower lobe BAL samples is presented in Fig. 2e. Droplet digital PCR via Taqman assay was used for independent validation of hsa-miR-451a expression levels. Correlation plot for hsa-miR-451a comparing platforms NanoString versus ddPCR reads is shown in Fig. 2f (*rho* = 0.7038, *P* = 0.002).

### Gene ontology (GO) and pathway analysis

**Fig. 3:**
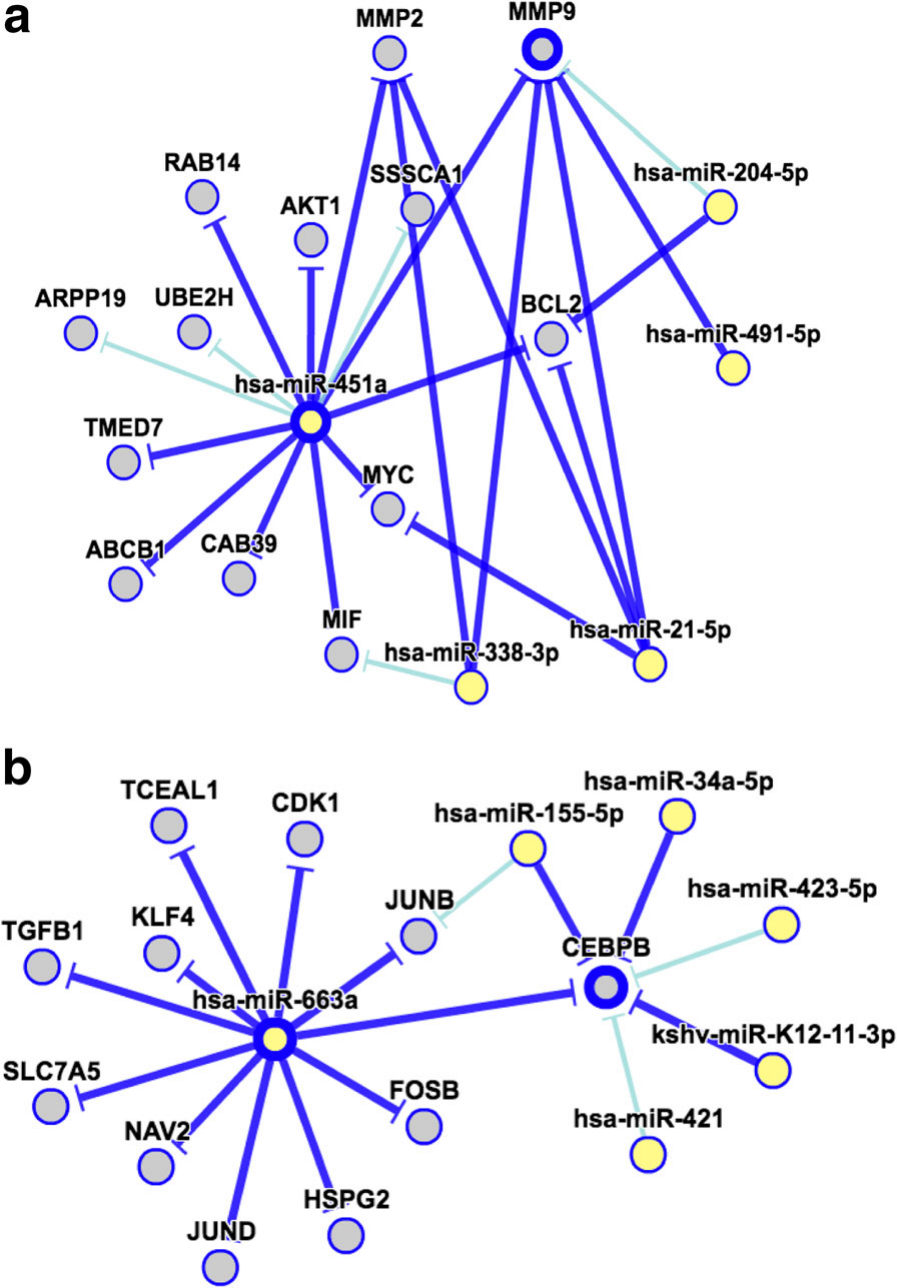
MicroRNA Gene Set Enrichment Networks. Mature hsa-miR-451a potentially targets 23 different genes, those with strong experimental evidence (miRTarBase) are shown by *dark blue extension lines* (Fig. 3**a**). Mature hsa-miR-663a potentially targets 105 different genes, with the top eleven with strong experimental evidence shown by *dark blue lines* (Fig. 3**b**)

Gene ontology was assessed using miRTarBase [21]. We performed in silico analysis of microRNAs identified differential expressed in BAL fluid to specifically identify potential gene targets of miR-451a and miR-663a. MicroRNA gene set enrichment networks (GSEN) identify 23 overall target genes for miR-451a, 10 of these with strong experimental evidence, including *MIF*, *MMP-2*, *MMP-9* and *AKT1* (Fig. 3a). Additionally, GSEN identified 105 possible miR-663a target genes, eleven of these with strong experimental evidence of interaction, including *CEBPβ* and *TGFβ1* (Fig. 3b). The top 10 experimentally validated gene targets of hsa-miR-451a and hsa-miR-663a along with the GO Biological Process key words for each are shown in Table 4.

**Table 4:** Validated gene targets of hsa-miR-451a & hsa-miR-663a and GO Biological Processes Key Words

Gene ID	Name	GO Biological Process Key Words
miR-451a gene targets		
MIF	(Macrophage migration inhibitory factor)	Immunity, inflammatory response, innate immunity
BCL2	(B cell lymphoma 2)	apoptosis
CAB39	(calcium binding protein 39)	cell cycle arrest
ABCB1	(Multidrug resistance protein 1)	transport
AKT1	(RAC-alpha serine/threonine-protein kinase)	apoptosis
MMP-2	(Matrix metalloproteinase 2)	angiogenesis, collagen degradation
MMP-9	(Matrix metalloproteinase 9)	collagen degradation
RAB14	(RAS-related protein Rab-14)	protein transport, transport
RAB5A	(RAS-related protein Rab-5A)	endocytosis, phagocytosis
IL6R	(IL6 Receptor)	cytokine mediated signaling pathway
miR-663a gene targets		
JUNB	(jun B proto-oncogene)	transcription, transcriptional regulation
JUND	(jun D proto-oncogene)	transcription, transcriptional regulation
KLF4	(Kruppel-like factor 4)	transcription, transcriptional regulation
FOSB	(FBJ murine osteosarcoma viral oncogene)	transcription regulation (AP1)
SLC7A5	(solute carrier family 7, member 5)	amino acid transport (lung)
NAV2	(neuron navigator 2)	helicase, hydrolase
CEBPB	(CCAAT/enhancer binding protein, beta)	transcription, transcriptional regulation
HSPG2	(heparin sulfate proteoglycan 2-perecan)	angiogenesis, (basement membrane)
CDK1	(cyclin-dependent kinase 1)	apoptosis, cell cycle & division, mitosis
TGFB	(transforming growth factor beta)	inflammatory response

## Discussion

Numerous pulmonary diseases occur with upper lobe predominance (ULP) including cystic fibrosis, smoking-related COPD, tuberculosis, and sarcoidosis [1]; however, no specific initiating cellular mechanisms driving these pathologies have been defined. The current dogma explaining ULP lung diseases is that gravity induces regional differences in perfusion-ventilation ratio, partial pressure of oxygen and lymphatic flow within the lung [1, 2] leading to regional differences in the host-to-pathogen response—upper lobe versus lower lobe in the lung. One potentially important difference comparing upper lobe vs. lower lobe is the variance in oxygen concentration between the regions, which equates to a decreasing hypoxic gradient from the apex (21% O_2_) to the base (10% O_2_) of the lung. Hypoxia has been shown to modulate multiple aspects of cell biology [5, 18]. Our goal was to investigate possible cellular mechanisms influencing regional differences in pulmonary cellular response associated with hypoxia. We hypothesized that the basal hypoxic gradient of the healthy lung may pre-program regional pulmonary cells to respond dissimilarly, conditional on the decreasing oxygen gradient concentrations. The reasoning behind gaining a better understanding of the regional pulmonary response is that this differential response could ultimately lead to differing innate immune responses to invading pathogens, pathogen colonization and/or disease progression. Studies presented here were conducted in the healthy lung to lay the groundwork for subsequent studies and a more precise interpretation of effects in the diseased lung. Additionally, we chose to focus primarily on microRNA, as these small non-coding RNA molecules are likely involved in a broad scope of transcriptional regulation and are increasingly being recognized as important modulators in respiratory disease [11, 12].

A number of recent studies have emerged in lung cancer, pulmonary fibrosis, chronic obstructive pulmonary disease (COPD), asthma, and cystic fibrosis, implicating microRNAs in the molecular pathogenesis of these lung diseases [22–24]. Additionally, hypoxia-dependent covariant microRNA biogenesis and secretion has also been demonstrated [25], where it was determined that the exosomal microRNA secretome is altered in response to hypoxia.

This study, as far as we know, is the first to report microRNA profiling from both regional alveolar macrophages and regional BAL fluid. Our approach is unique in that it provides a direct comparison of the epigenetic molecular signature of the upper lobe vs. the lower lobe from each of these primary human bio-specimen sources.

Our initial in vitro experiments demonstrating a hypoxia-associated early response, and regional differential IL-8 and TNF-α secretion from AM, proved interesting, demonstrating hypoxia-associated effects in upper lobe vs. lower lobe AM. This led us to wonder about the broader extent of zonal AM gene modulation with oxygen stress. We focused the remainder of our effort at the level of epigenetic transcriptional regulation. To examine more closely the potential cellular mechanisms involved here, we chose to investigate transcriptional regulation via a high-throughput microRNA profiling platform. In addition, we chose a 24-h hypoxia, as this more closely simulates the in vivo O_2_ steady state of AM in lung.

As a first approach, we examined the microRNA content of AM and identified a number of microRNAs that play roles related to innate immunity, inflammation or extracellular matrix remodeling including: miR-93-5p, miR-22-3p and miR-4454. Potential targets identified via miRTarBase [21] of miR-93-5p include *PTEN* [26] and *Il-8* [27], of miRNA-22-5p include *PPARA* and *BMP7* [28] and *HMGB1* [29], and of miR-4454 include *TRAM2* and *CCR4*. MicroRNA regulation of cellular processes is a complicated issue as each specific miRNA can have a multitude of mRNA targets [13]. The one overlap we noted between our protein data and microRNA data was the observation of the increase of miR-93-5p expression seen in lower lobe AM is consistent with our observed decreased of IL-8 secretion by AM in vitro.

Next, we wanted to determine if we could measure a regional difference in microRNA from the exosomes/ microvesicles secreted or shed into the BAL fluid. Only two studies to date have examined microRNA in human BAL fluid [30, 31] and neither addressed the regional distribution of microRNAs within the lungs. Interestingly, miRNA content of regional BAL fluid in this study shows divergence of several microRNAs that could prove to be of importance in inflammation within the lung, namely miR-451a and miR-663. miRTarBase gene set enrichment network analysis has identified several experimentally validated targets of miR-451a that are certainly intriguing in the context of innate immunity including: *MIF* [32–34] and *MMP-2* and *MMP-9* [35, 36]. MIF could potentially alter macrophage movement regionally and MMP-2 and MMP-9 may alter extracellular matrix structural integrity, ultimately influencing altered fibrosis regionally. It’s not readily apparent how the differential levels of miR-451a, or other microRNAs we identified effect their potential targets in vivo. Likewise, it has also not been established the exact validated targets ultimately effected in this setting.

It is also worth noting at this point that the zonally divergent microRNAs detected in BAL fluid did not overlap with the hypoxia-divergent microRNAs detected within macrophages. This may simply indicate that the cell source(s) of miRNAs in BAL fluid is a cell-type other than the alveolar macrophage, consistent with numerous studies having demonstrated cell-type-specific microRNA expression [37–39].

An emerging area of interest in eukaryotic biology is the subject of trans-kingdom cross-talk, whereby small molecules produced by the host influence a symbiont or pathogen. MicroRNAs are at the forefront of these types of studies. It is possible that some of the microRNAs detected here may be functioning in a host-pathogen trans-kingdom cross-talk role. For instance, a BLAST of the hsa-miR-451a mature sequence on the fungal and oomycete genomics database: FungiDB, reveals 100% sequence homology to gene Afu5g11540 - localized to the mitochondrial ribosome of *Aspergillis fumigatus. Aspergillis fumigatus* is one of the main fungal species found in cystic fibrosis airways [40]. If hsa-miR-451a does influence Aspergillis fumigatus growth, it is easy to see how differing levels of this non-coding RNA regionally in the human lung may alter the host-pathogen response. Future studies to address this topic in the context of a lung disease such as cystic fibrosis could prove very useful in better understanding the host-pathogen relationship.

Our current study has a number of strengths. The availability and analysis of regional lung macrophages and BAL fluid microRNA content makes this pulmonary study unique. Additionally, the identification of divergent zonal microRNA profiles coupled with the use of in silico analysis by miRTarBase allows for development of new hypotheses linking experimentally validated microRNA targets and innate immunity pathways.

This work, however, also has a number of limitations. Firstly, this is a descriptive study and as such does not address the specific intracellular or intercellular regulatory mechanisms of microRNA within the lung. Additionally, identifying the specific cell source(s) of the microRNA in BAL fluid was beyond the scope of this study.

## Conclusions

In summary, we present evidence that the microRNA complement of the upper lobe of the lung is different than the lower lobe and suggest that hypoxia within the lung may at least in part be responsible for these molecular differences. We have demonstrated that hypoxia can drive alterations in microRNA content in alveolar macrophages potentially influencing the inflammatory response. Additionally, basal microRNA content of upper lobe BAL fluid is different than lower lobe BAL fluid, indicating an association with the natural hypoxic gradient that exists from the apex to the base of the lung.

Ultimately, it remains to be determined if the specific microRNAs identified in this study, miR-93, miR-4454, miR-451a or miR-663a, function intracellularly, in a paracrine or endocrine manner in cell-to-cell communication, or are involved in trans-kingdom crosstalk with potential pathogens, or some combination of these processes.

## Additional files

Additional file 1: Online supplemental methods. (DOCX 97 kb)

Additional file 2: Figure S1. Exosomes/microvesiclescontaining microRNAs were isolated from Bronchoalveolar Lavage (BAL) Fluid. Agilent Bio-Analyzer was used to quantitate microRNA recovery. 1 μl per sample run on BioAnalyzer 2100 with the Small RNA Chip kit. MIcroRNA seen at 20–40 nt, additional peaks in electropherogram represent tRNA and other small RNAs. (RLL: right lower lobe; RUL: right upper lobe). (TIFF 563 kb)

Additional file 3: Figure S2. Correlation of NanoString nCounter microRNA assay technical replicates. Technical replicates of alveolar macrophage total RNA were run on the NanoString nCounter microRNA assay. Strong correlation was seen between replicates (*rho* = 0.987 *P* < 0.0001). (PDF 67 kb)

## Abbreviations

AM: Alveolar macrophage
BAL: Bronchoavelolar lavage
CF: Cystic fibrosis
ddPCR: Droplet digital polymerase chain reaction
GO: Gene ontology
GSEN: Gene set enrichment network
miRNA: MicroRNA
