# Supplementary material for: Pulmonary microRNA profiling: implications in upper lobe predominant lung disease
Source: Clin Epigenetics. 2017 May 30;9:56. doi: 10.1186/s13148-017-0355-1 (PMC5450072; doi:10.1186/s13148-017-0355-1)
Supplement: Supplementary file 1 — Online supplemental methods. (DOCX 97 kb) [file 13148_2017_355_MOESM1_ESM.docx]

**ONLINE SUPPLEMENT**

**Pulmonary MicroRNA Profiling: Implications in Upper Lobe Predominant Lung Disease**

David A. Armstrong, Amanda B. Nymon, Carol Ringelberg, Corina Lesseur, Haley F. Hazlett, Louisa Howard, Carmen J. Marsit and Alix Ashare

**Supplemental Methods**

**Sample Collection**

Following written informed consent, subjects underwent flexible bronchoscopy. After local anesthesia with viscous lidocaine to the posterior pharynx and intravenous sedation, a bronchoscope was inserted trans-orally and advanced through the vocal cords. Bronchoalveolar lavage (BAL) fluid and alveolar macrophage (AM) samples were obtained from tertiary airways in the right upper, middle and/or lower lobes (RUL, RML and RLL, respectively). BAL was performed sequentially in the RUL, RML, and RLL with 20 ml of sterile saline followed by 10 ml of air. In all subjects BAL fluid return was between 12 ml and 15 ml from the RUL and between 10 ml and 12 ml from the RLL.

**Total RNA / MicroRNA Extraction, Purification and Quantification**

Total RNA was extracted from AM using Zymo Quick-RNA Mini-prep Kit (Irvine, CA). Cell-free microRNA from BAL fluid was obtained using Norgen Urine Exosome RNA Isolation Kit (Norgen Biotek Corp., Thorold, ON, Canada) from a 0.3 ml volume of cell-free BAL fluid. All protocols were performed according to manufacturer’s instructions. BAL microRNA was purified and concentrated using Amicon Ultra 0.5 columns (Millipore, Billerica,MA) (Erroll Reuckert, NanoString personal communication). Briefly, eluted microRNA was brought to a total volume of 420ul in nuclease-free water, column/collection tube loaded and centrifuged at 14,000 x g for 20 minutes at 20C. Column was placed in a fresh collection tube in inverted position and centrifuged 8,000 x g 2 minutes at 20C. Recovered miRNA was concentrated on Speed Vac (ThermoFisher Scientific, Waltham, MA) 15-20 minutes at 20 C. Total RNA was quantified using a Qubit 3.0 fluorometer (ThermoFisher Scientific, Waltham,MA). MicroRNA was quantified on the Agilent Bioanalyzer 2100 (Agilent Technologies, Santa Clara, CA) using the Small RNA Chip assay (Electropherogram profiles and miRNA concentrations (picograms per microliter(pg/ul) are representatively shown in Additional figure1.

**Nanostring microRNA Assays and Droplet digital PCR**

RNA samples were aliquoted and stored at -80 C and samples were thawed only once for expression analysis. The digital multiplexed NanoString nCounter human v3 microRNA expression assay (NanoString Technologies, Seattle,WA) was performed according to manufacturer’s instructions with total RNA or miRNAs extracted as above. Briefly, 3-10 ng microRNA (BAL fluid) or 100 ng total RNA (AM) samples were prepared by ligating a specific miR-tag onto the 3’ end of each mature miR followed by an overnight hybridization (65 C) to nCounter Reporter and Capture probes. Excess Reporter and Capture probes are washed away using the automated nCounter sample prep station and probe/target complexes are aligned and immobilized in the nCounter Cartridge. Cartridges are then placed in the nCounter digital analyzer for data collection. nSolver Analysis software (NanoString) (V3.0) was used for data analysis including background correction by subtracting the mean of the six negative controls included on the NanoString platform and normalization using the average geometric mean of the top one hundred probes detected. Detectable probes is defined as read counts > 20 in > 50% of the samples after background subtraction and normalization. Technical replicates showed strong correlation (*rho* = 0.987 *P* <0.0001)(Additional figure 2).

First strand reaction was performed with TaqMan microRNA reverse transcription kit (Life Technologies, Grand Island, NY) following manufacturer’s protocols. Droplet digital PCR (ddPCR) was performed with the QX100 Droplet Digital PCR system as follows: 20 μL of the reaction mixture containing 8 μL of cDNA solution, 10 μL of digital PCR^TM^ Supermix (Bio-Rad, Hercules, CA), and 1 μL of Taqman primer/probe mix (Life Technologies) and DEPC H_2_O was loaded into a plastic cartridge with 70 μL of QX100 Droplet Generation oil and then placed into the QX100 Droplet Generator. The droplets generated from each sample were transferred to a 96-well PCR plate (Eppendorf, Germany). PCR amplification was carried on a Veriti thermal cycler (Life Technologies) at 95°C for 10 min, followed by 40 cycles of 95°C for 30 seconds and 60°C for 1 min, then 1 cycle of 98°C for 10 min, ending at 4°C. The plate was then loaded on Droplet Reader for analysis. Absolute quantification of each miRNA was calculated from the number of positive counts per panel using the Poisson distribution. The quantification of the target miRNAs was presented as the number of copies/μL of PCR mixture.

**Enzyme-linked Immunosorbant Assays**

Human CXCL8 /IL8 and TNFα were measured from starting volume of 100 ul of conditioned media with DuoSet ELISA Kit from R & D Systems (Minneapolis, MN) according to manufacturer’s instructions.

**Electron Microscopy**

Exosomes / microvesicles (E/Mv) were isolated via affinity resin of the Urine Exosome RNA Isolation kit (Norgen Biotek, Ontario,Canada). Norgen resin was rinsed with 1x PBS for electron microscopy to release E/Mv. The “wash” method was used for negative staining: Formvar-coated 300 mesh Cu grids (Electron Microscopy Science) were floated on top of 20ul drops of exosomes / microvesicles for 4 minutes; then the grid was placed on 5 consecutive drops of 2% Uranyl Acetate (UA) and left on 5^th^ drop of 2% UA for 1 to 1.5 minutes. All TEM images were taken at 100 kV on a JEOL TEM1010 equipped with a XR-41B AMT digital camera and capture engine software (AMTV540; Advanced Microscopy Techniques).

**Gene Ontology**

Gene ontology (GO) and pathway analysis were performed using MirTarBase. This newly developed microRNA database has accumulated more than 360,000 miRNA-target interactions (MTIs), while containing the largest amount of validated MTIs. MirTarBase data base provides information related to specific microRNAs including: sequence information, target genes, validated evidences and methods used, gene enrichment sets based on functional analysis and network diagrams.
