# Supplementary figures and images for: Pulmonary microRNA profiling: implications in upper lobe predominant lung disease
Source: Clin Epigenetics. 2017 May 30;9:56. doi: 10.1186/s13148-017-0355-1 (PMC5450072; doi:10.1186/s13148-017-0355-1)

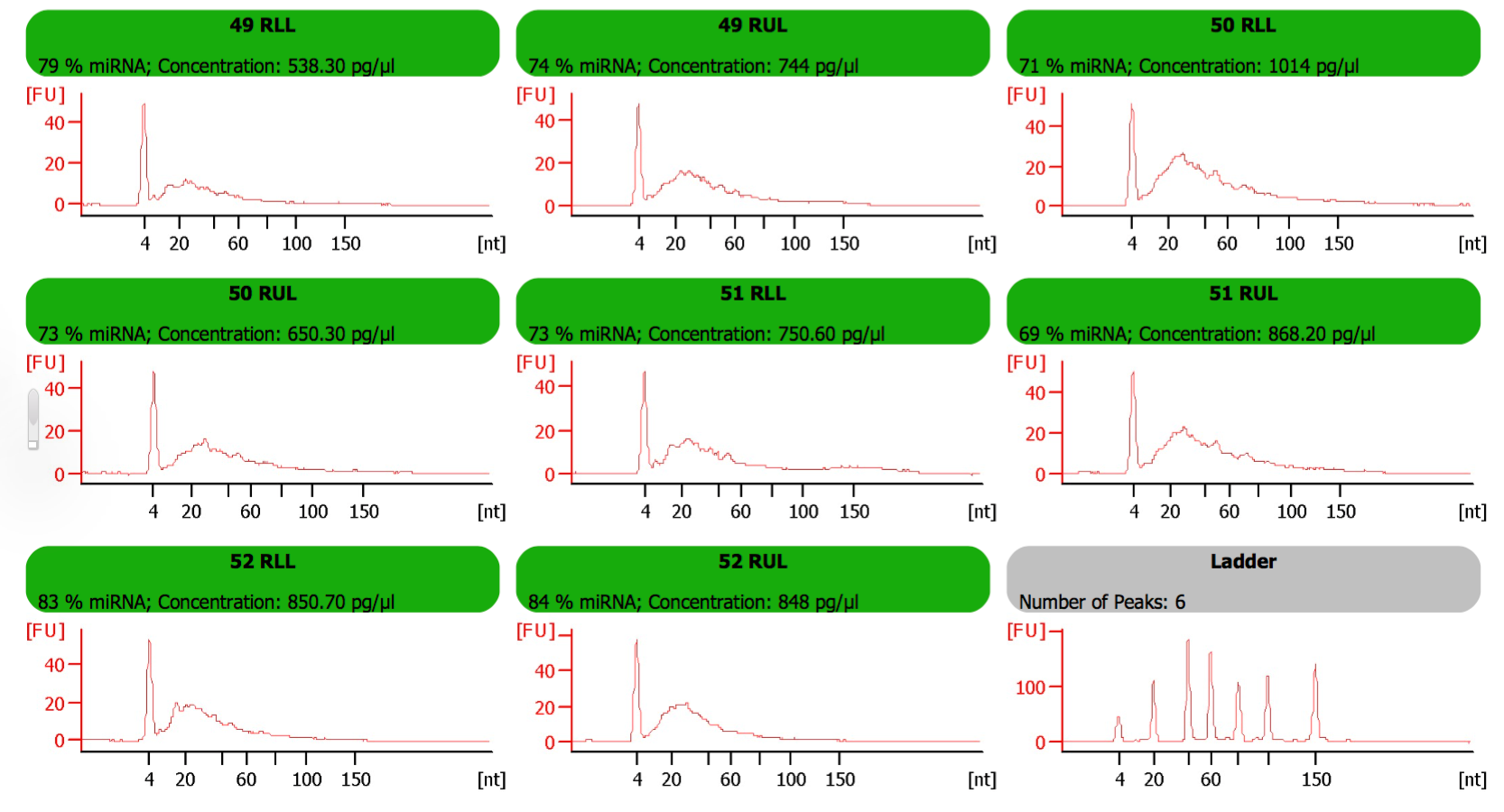

Supplement: Supplementary file 2 — Exosomes/microvesiclescontaining microRNAs were isolated from Bronchoalveolar Lavage (BAL) Fluid. Agilent Bio-Analyzer was used to quantitate microRNA recovery. 1 μl per sample run on BioAnalyzer 2100 with the Small RNA Chip kit. MIcroRNA seen at 20–40 nt, additional peaks in electropherogram represent tRNA and other small RNAs. (RLL: right lower lobe; RUL: right upper lobe). (TIFF 563 kb) [file 13148_2017_355_MOESM2_ESM.tiff]

MicroRNA Reads Alveolar Macrophage  
Total RNA Technical Replicate 1

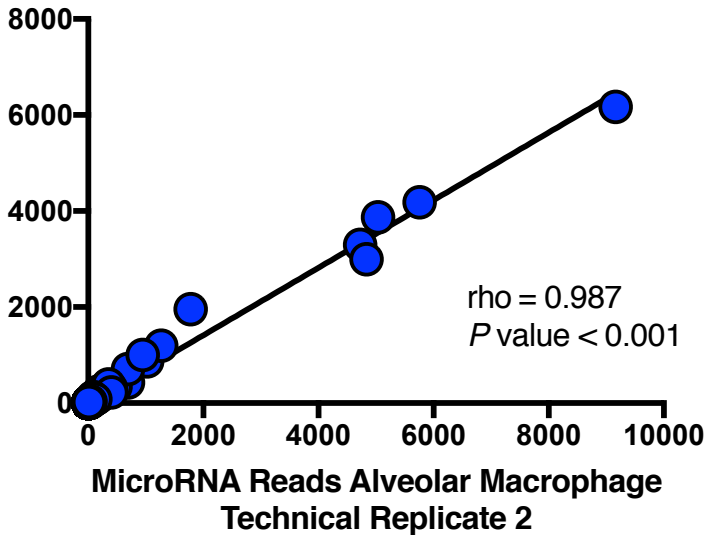

Supplement: Supplementary file 3 — Correlation of NanoString nCounter microRNA assay technical replicates. Technical replicates of alveolar macrophage total RNA were run on the NanoString nCounter microRNA assay. Strong correlation was seen between replicates (rho = 0.987 P < 0.0001). (PDF 67 kb) [file 13148_2017_355_MOESM3_ESM.pdf]
